# Contribution of NADPH-cytochrome P450 Reductase to Azole Resistance in *Fusarium oxysporum*

**DOI:** 10.3389/fmicb.2021.709942

**Published:** 2021-09-14

**Authors:** Dan He, Zeqing Feng, Song Gao, Yunyun Wei, Shuaishuai Han, Li Wang

**Affiliations:** ^1^Department of Pathogenobiology, Jilin University Mycology Research Center, Key Laboratory of Zoonosis Research, Ministry of Education, College of Basic Medical Sciences, Jilin University, Changchun, China; ^2^Beijing ZhongKaiTianCheng Bio-technonogy Co. Ltd., Beijing, China

**Keywords:** *Fusarium oxysporum*, NADPH-cytochrome P450 reductase, azole resistance, *Agrobacterium tumefaciens*-mediated transformation, ergosterol biosynthesis, antifungal susceptibility

## Abstract

*Fusarium* species exhibit significant intrinsic resistance to most antifungal agents and fungicides, resulting in high mortality rates among immunocompromised patients. Consequently, a thorough characterization of the antifungal resistance mechanism is required for effective treatments and for preventing fungal infections and reducing antifungal resistance. In this study, an isolate of *Fusarium oxysporum* (wild-type) with broadly resistant to commonly antifungal agents was used to generate 1,450 T-DNA random insertion mutants *via Agrobacterium tumefaciens*-mediated transformation. Antifungal susceptibility test results revealed one mutant with increased sensitivity to azoles. Compared with the resistant wild-type, the mutant exhibited low MICs to KTZ, ITC, VRC, POS, and PCZ (0.125, 1, 0.06, 0.5, and 0.125μg/ml, respectively). The T-DNA insertion site of this mutant was characterized as involving two adjacent genes, one encoding a hypothetical protein with unknown function and the other encoding the NADPH-cytochrome P450 reductase, referred as CPR1. To confirm the involvement of these genes in the altered azole susceptibility, the independent deletion mutants were generated and the *Cpr1* deletion mutant displayed the same phenotypes as the T-DNA random mutant. The deletion of *Cpr1* significantly decreased ergosterol levels. Additionally, the expression of the downstream *Cyp51* gene was affected, which likely contributed to the observed increased susceptibility to azoles. These findings verified the association between *Cpr1* and azole susceptibility in *F. oxysporum*. Furthermore, this gene may be targeted to improve antifungal treatments.

## Introduction

*Fusarium* species, which are well-known filamentous ascomycetous fungi, include many agriculturally important plant pathogens and opportunistic pathogens of humans and other animals (Ma et al., 2013; Al-Hatmi et al., 2016; Tupaki-Sreepurna and Kindo, 2018; Zhao et al., 2021). *Fusarium* species usually cause local infections, including fungal keratitis, which often leads to blindness. However, over the last few decades, the number of dangerously invasive infections has increased in immunocompromised individuals, especially cancer patients with prolonged neutropenia and patients with hematological disorders. These infections can spread to the lungs, heart, liver, kidneys, and central nervous system (Tupaki-Sreepurna and Kindo, 2018; Lockhart and Guarner, 2019; Batista et al., 2020; Hof, 2020). As emerging fungal pathogens, some *Fusarium* species, such as *Fusarium oxysporum* and *Fusarium solani*, are now among the most common pathogenic molds associated with significant morbidity and mortality, behind only *Aspergillus* and Mucorales molds (Miceli and Lee, 2011; Guarro, 2013; Tortorano et al., 2014; Al-Hatmi et al., 2016; Lockhart and Guarner, 2019; Hof, 2020).

Antifungal therapy is necessary for successful disease management. However, because of intrinsic resistance and selection pressure, infections caused by *Fusarium* species are relatively difficult to treat. Most species of this genus are typically resistant to a broad range of antifungal agents developed for clinical use, including azoles, polyenes, and echinocandin. They are also minimally susceptible to agricultural fungicides (Azor et al., 2007; Miceli and Lee, 2011; Ma et al., 2013; Ribas et al., 2016; Sharma and Chowdhary, 2017; Batista et al., 2020; Hof, 2020). *In vitro* studies have indicated amphotericin B and echinocandin are relatively ineffective for controlling *Fusarium* species, whereas triazoles, such as voriconazole and posaconazole, are effective against almost 50% of isolates (Azor et al., 2007; Miceli and Lee, 2011; Tortorano et al., 2014). Therefore, the mechanisms underlying the antifungal resistance of *Fusarium* species must be characterized.

Most of the studies on the antifungal resistance of pathogenic fungi conducted to date have focused on the genera *Candida* and *Aspergillus*. There has been relatively little related research regarding *Fusarium* species, with most studies examining the susceptibility of the species to antifungal agents. The few studies analyzing resistance mechanisms have mostly involved plant pathogens and investigations of the changes in the amino acid sequence encoded by the *Fks1* gene or the effects of overexpressing the *Cyp51* gene or the genes encoding ABC efflux pumps (Katiyar and Edlind, 2009; Abou Ammar et al., 2013; Zhang et al., 2021; Zhao et al., 2021).

To identify genes related to the antifungal resistance of *Fusarium* species, *Agrobacterium tumefaciens*-mediated transformation (ATMT) was used to construct T-DNA random insertion mutants. The 1,450 generated mutants from a broadly resistant isolate of *F. oxysporum* included FOM1123, which exhibited altered susceptibility to azoles. We functionally characterized the genes interrupted by the T-DNA insertion and clarified their regulatory roles related to antifungal resistance.

## Materials and Methods

### Strains and Plasmids

Wild-type *F. oxysporum* JLCC31768, which was originally isolated from a patient with fungal keratitis in Jilin province, China, was used to construct T-DNA random insertion mutants. The antifungal susceptibility test (AFST) results revealed it is broadly resistant to different azoles, amphotericin B, and caspofungin commonly used in clinical settings (Table 1).

**Table 1:** Antifungal susceptibility test results for the wild-type *Fusarium oxysporum* and the mutants (MIC, μg/ml).

	KTZ	FLU	ITC	VRC	POS	PCZ	AMB	CFG
Wild-type	8	>64	>16	4	4	8	1	>16
FOM1123	0.125	>64	1	0.06	0.5	0.125	1	>16
*Δ*HPG	8	>64	>16	4	4	8	1	>16
*Δ*CPR1	0.125	>64	1	0.06	0.5	0.125	1	>16
*Δ*CPR2	8	>64	>16	4	4	8	1	>16
*Δ*CPR3	8	>64	>16	4	4	8	1	>16
*Δ*CPR4	8	>64	>16	4	4	8	1	>16

Plasmids pXEH and pXEN (containing neomycin and kanamycin resistance tags) as well as *A. tumefaciens* Agr0 and AgrN (containing pXEN) were used to generate *F. oxysporum* mutants. All strains and plasmids were preserved at the Jilin University Mycology Research Center (Jilin, China).

### Construction of Random Insertion Mutants

Antifungal resistance tests indicated that wild-type *F. oxysporum* is sensitive to geneticin (G418). Accordingly, geneticin was selected as a resistance tag. The geneticin phosphotransferase II gene (*Neo*) mediating G418 resistance was ligated to pXEH to construct the pXEN recombinant plasmid. *A. tumefaciens* Agr0 cells were transformed with pXEN to obtain the AgrN strain, which was used for ATMT.

The *F. oxysporum* T-DNA insertion mutants were generated as previously described (Fan et al., 2016). Briefly, fungal spores (1×10^4^CFU/ml) were mixed with an equal volume (1ml) of AgrN cells (OD_600nm_=0.8). A Millipore filter was placed on the surface of solid induction medium containing 200μm acetosyringone. A 200μl aliquot of the spore–AgrN mixture was spread evenly on the filter. After incubating for 48h at 25°C in darkness, the filter was transferred to selection medium (PDA containing 200μm cefotaxime sodium and 100μg/ml G418) and incubated at 25°C. The mutants were used to inoculate PDA slants in tubes.

Genomic DNA was extracted from randomly selected mutants using the TIANgel Rapid Mini Plasmid Kit (Tiangen Biotech, Beijing, China) for a PCR amplification using the neoF and neoR primers specific for the *Neo* gene (Table 2). The amplified products were sequenced by Comate Bioscience Co., Ltd (Jilin, China), after which the sequences were analyzed to determine whether the T-DNA was inserted into the *F. oxysporum* genome. After multiple transformations, many T-DNA insertion mutants were preserved for further research.

**Table 2:** Primers used to generate the T-DNA insertion mutants and deletion mutants.

Primer name	Nucleotide sequence (5' to 3')
neoF	ATCTCCTGTCATCTCACCTTGCTC
neoR	GTCTCCTTCCGTGTTTCAGTTAGC
LB1	GGGTTCCTATAGGGTTTCGCTCATG
LB2	CATGTGTTGAGCATATAAGAAACCCT
LB3	GAATTAATTCGGCGTTAATTCAGT
RB1	GGCACTGGCCGTCGTTTTACAAC
RB2	AACGTCGTGACTGGGAAAACCCT
RB3	CCCTTCCCAACAGTTGCGCA
AD1	TGAGNAGTANCAGAGA
AD2	AGTGNAGAANCAAAGG
AD3	CATCGNCNGANACGAA
AD4	CAAGCAAGCA
4LTf	CCCAAGCTTCATGTCGTTCAGTATCTCGCTATAAGCAT
4LTr	CGCGGATCCGTGTCCAATTCACTTTCGGGTTT
4RTf	CGGGGTACCCATTAACGAACGCGACGACCT
4RTr	CGGACTAGTCAGACATAGAAATAAGCCTTCTG
3LTf	CCGGAATTCGCCTGAACAGCAACATGTAAGAGTT
3LTr	CGGGGTACCGAAACTTGCCAATTGGAACCT
3RTf	TGCTCTAGAGTATTCCCGCGATACAGCCCAGAT
3RTr	CGCGTCGACCCCCTCAAATTATAGAAAACTTGTGC

### Antifungal Susceptibility Testing

The AFST was performed using the CLSI broth microdilution method as described in M38-Ed3 (Clinical and Laboratory Standards Institute, 2017). The following antifungal agents, including azole fungicides, were tested: fluconazole (FLU; NICPBP, Beijing, China), itraconazole (ITC; Sigma, St. Louis, MO, United States), voriconazole (VRC; Sigma), posaconazole (POS; Sigma), amphotericin B (AMB; Sigma), caspofungin (CFG; Meilunbio, Dalian, China), ketoconazole (KTZ; NICPBP), and propiconazole (PCZ; NICPBP). The antifungal agents were diluted 10 times (2-fold dilutions) for the following concentration ranges: FLU, 0.125–64μg/ml; ITC, VRC, POS, AMB, CFG, KTZ, and PCZ, 0.03–16μg/ml. As recommended by CLSI, *Candida krusei* ATCC6258 and *Candida parapsilosis* ATCC22019 were used as quality control strains. The MIC endpoint for AMB was defined as the lowest concentration with 100% growth inhibition relative to the antifungal-free control. For the other antifungal agents, the MICs were defined as the lowest concentration with a prominent decrease in growth (almost 50%) relative to the control. After a comparison with the wild-type *F. oxysporum*, the mutants with altered antifungal susceptibility were selected.

### Bioinformatics Analysis of Related Genes in Mutants With Altered Antifungal Susceptibility

The sequences flanking the inserted T-DNA were amplified by touchdown-TAIL-PCR using previously described primers (Table 2; Gao et al., 2016). To determine the insertion sites, the flanking sequences were aligned with the *F. oxysporum* f. sp. *lycopersici* genome (taxid: 426428) using the Basic Local Alignment Search Tool (BLAST).^1^ Relevant information regarding the interrupted genes was obtained from the NCBI, KEGG, and UniProtKB databases.

### Construction of Deletion Mutants

By exploiting homologous genetic recombination, the genes interrupted by the T-DNA insertion, including *Hpg* as well as *Cpr1* and its homologs (*Cpr2*, *Cpr3*, and *Cpr4*), were targeted using specific primers (Table 2) to produce *F. oxysporum* deletion mutants. The *Hpg*, *Cpr1*, *Cpr2*, *Cpr3*, and *Cpr4* genes were replaced by *Neo* in pXEN, which was then inserted into Agr0 cells. The *Δ*HPG, *Δ*CPR1, *Δ*CPR2, *Δ*CPR3, and *Δ*CPR4 deletion mutants were generated by ATMT.

### Analysis of the Biological Characteristics of the Deletion Mutants

To compare colony morphologies, PDA medium was inoculated with the wild-type *F. oxysporum* and the deletion mutants and then incubated at 25°C for 5days. Slide cultures were prepared for these strains and then examined using a microscope after lactophenol cotton blue staining. Additionally, the susceptibility of the deletion mutants to antifungal agents was tested as described in M38-Ed3 (Clinical and Laboratory Standards Institute, 2017).

### Determination of Ergosterol Content

Fungal spores (1×10^6^CFU) were used to inoculate 100ml PDB medium, which was then incubated at 25°C for 48h with shaking (180rpm). In the antifungal treatment group, VRC (amount corresponding to the 0.5 MIC) was added after 24h. Next, 100mg mycelia were collected and resuspended in 5ml deionized water before being disrupted for 20min using the Scientz-IID ultrasonic cell disrupter (SCIENTZ, Ningbo, China). A 5ml aliquot of the solution was mixed with 20ml ether. The absorbance of the resulting extract was measured at 281.5nm. The ergosterol content was calculated using a standard curve.

### Expression Analysis of Genes Involved in Ergosterol Biosynthesis

Mycelia were disrupted by grinding in liquid nitrogen. Total RNA was extracted from the ground material using the RNAiso Plus kit (Takara, Shiga, Japan). The RNA concentration was determined using the NanoDrop One spectrophotometer (Thermo Fisher, San Jose, CA, United States). The RNA served as the template for synthesizing cDNA using the HiScript II Q RT SuperMix for qPCR (Vazyme, Nanjing, China). Quantitative real-time PCR was performed using the AceQ qPCR SYBR Green Master Mix (Vazyme), gene-specific primers (Table 3), and the 7500 Fast Real-Time PCR System (Applied Biosystems, Foster City, CA, United States). The expression levels of genes related to ergosterol synthesis (e.g., *Cpr*, *Cytb5*, and *Cyp51*) were normalized against the expression of the 18S rRNA housekeeping gene. Relative gene expression levels were calculated according to the 2^−ΔΔCT^ method (Livak and Schmittgen, 2001).

**Table 3:** Primers used for the quantitative real-time PCR analysis.

Primer Name	Nucleotide Sequence (5' to 3')	Purpose
18Sqf	CGCCAGAGGACCCCTAAAC	normalization
18Sqr	ATCGATGCCAGAACCAAGAGA	
CPR1qf	TGCAATCCCGCAATTGAGCC	FOXG 08274
CPR1qr	TCAGAACAGCTACGGAATGCCA	
CPR2qf	TGAGTTGACATCCCGAGCCA	FOXG_07461
CPR2qr	TCTCCAGAGAGGCCAAGCAA	
CPR3qf	GGTATTGATAACTCGCCTCTTC	FOXG_03206
CPR3qr	GTTGCTTGCTGTCACCATTA	
CPR4qf	GCAGCCGATAACCTACAC	FOXG_04834
CPR4qr	CCAGGACCAACCATAAGAATAG	
Cytb5qf	GACGGCAAGACAGTGAATCGC	FOXG_03180
Cytb5qr	CCGAGGGAGAGATTGGCAAGG	
CYP51Aqf	TCACCCTTCTCATGGCTGGAC	FOXG_11545
CYP51Aqr	GGGAGAACACCATCAGCACTCA	
CYP51Bqf	ATTGCTCTCCTCATGGCTGGC	FOXG_00394
CYP51Bqr	GGGAGGCAAATCAGCACCGA	
CYP51Cqf	GAATGGACAGGTTATCAAGGAG	FOXG_13138
CYP51Cqr	GGAGAAGCGAGGAGTGTAT	

### Statistical Analysis

Data are presented as the mean±standard deviation of at least three replicated measurements. Differences between the VRC-treated and untreated samples were evaluated by a one-way ANOVA followed by the *T* test using SPSS 2.0 (*p*<0.05 was set as the threshold for significance).

## Results

### Construction of T-DNA Random Insertion Mutants

**Figure 1:**
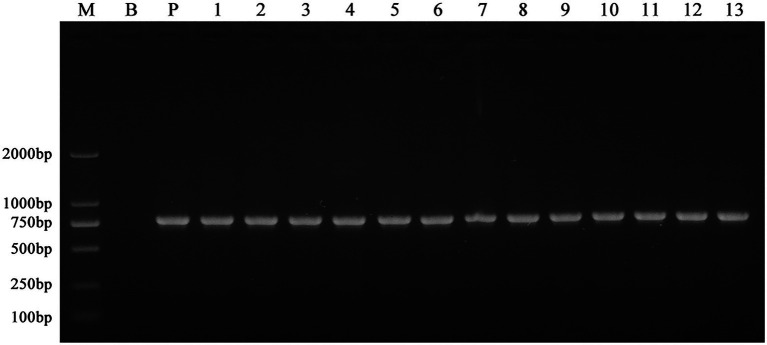
PCR amplification of the *Neo* gene in the wild-type *F. oxysporum* and the T-DNA insertion mutants. Genomic DNA of the mutants grown on the selection medium containing G418 was amplified using the neoF and neoR primers. All the mutants generated in this study produced a specific amplicon (approximately 700~750bp). Here, only showed the results of 13 different mutants selected randomly. These indicated the T-DNA containing the G418 resistance tag was inserted into the *F. oxysporum* genome. M: Trans 2 K marker; B: wild-type; P: pXEN; and lanes 1–13: 13 mutants with different T-DNA insertion.

Using an established ATMT system, wild-type *F. oxysporum* was transformed, with an efficiency of 250 mutants per 10^4^CFU. The PCR amplification of the *Neo* gene resulted in a single specific amplicon (approximately 700~750bp) for all T-DNA random mutants generated in this study (Figure 1). The sequenced fragment was 99% similar to the *Neo* gene, indicating the T-DNA containing the G418 resistance tag was inserted into the *F. oxysporum* genome. After multiple transformations, 1,450 mutants were obtained.

### Identification of Mutants With Altered Antifungal Susceptibility

The AFST results for the 1,450 confirmed mutants revealed one mutant (FOM1123) with altered antifungal susceptibility. More specifically, this mutant exhibited significantly increased susceptibility to azoles (except for FLU) with low MICs to KTZ, ITC, VRC, POS, and PCZ (0.125, 1, 0.06, 0.5, and 0.125μg/ml, respectively), compared with the resistant wild-type with high MICs (8,>16, 4, 4, and 8μg/ml, respectively). In contrast, its susceptibility to the polyene AMB and the echinocandin CFG was unchanged (Table 1). These observations suggested that the gene interrupted by T-DNA insertion in this mutant might be related to azole resistance in the wild-type *F. oxysporum*.

### Bioinformatics Analysis of Related Genes in the Mutant FOM1123

**Figure 2:**
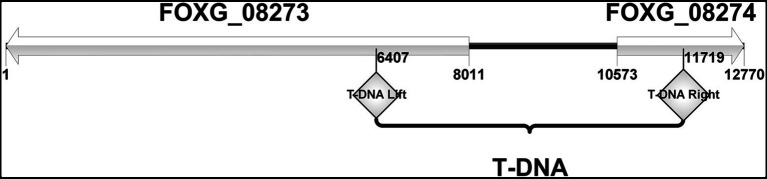
The site of T-DNA insertion of mutant FOM1123. It was characterized as involving 2 adjacent genes, FOXG_08273 and FOXG_08274. The inserted T-DNA replaced a 5,312bp sequence between the initiation regions of these two genes, from 2,932,119bp to 2,937,431bp on *F. oxysporum* chromosome 2.

The sequences flanking the inserted T-DNA in the mutant FOM1123 were amplified by touchdown-TAIL-PCR and sequenced. The subsequent BLAST analysis of the *F. oxysporum* genome indicated the T-DNA fragment replaced a 5,312bp sequence from 2,932,119bp to 2,937,431bp on chromosome 2 between the initiation regions of genes FOXG_08273 and FOXG_08274 (Figure 2). The FOXG_08273 gene encodes a hypothetical protein (HPG) comprising 2,548 amino acids. Its function is unknown, and no homologs were identified. The FOXG_08274 gene encodes an NADPH-cytochrome P450 reductase (CPR1) consisting of 692 amino acids. This gene, which is related to ergosterol biosynthesis, contributes to the delivery of electrons to P450 enzymes, similar to *Cytb5* (cytochrome *b5*). It has three homologs, namely, FOXG_07461 (*Cpr2* on chromosome 4), FOXG_03206 (*Cpr3* on chromosome 8), and FOXG_04834 (*Cpr4* on chromosome 7).

### Biological Characteristics of Deletion Mutants

**Figure 3:**
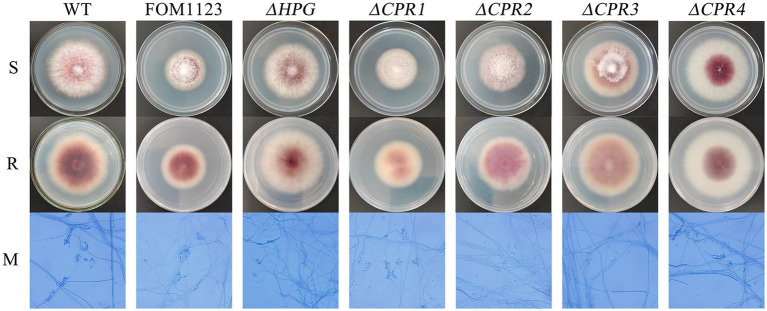
Colony and microscopic morphology of the wild-type *F. oxysporum* and the mutants. Strains cultured on PDA medium were incubated at 25°C for 5days. Slide cultures were examined after lactophenol cotton blue staining (PDA, 25°C, 3days, 400×magnification). S: surface of colony, R: reverse of colony, and M: micromorphology.

Compared with the wild-type *F. oxysporum*, the T-DNA insertion mutant FOM1123 and the deletion mutants *Δ*HPG, *Δ*CPR1, *Δ*CPR2, *Δ*CPR3, and *Δ*CPR4 had no obvious differences regarding colony and microscopic morphological characteristics, including mycelial growth, pigment production, spore germination, and spore structure (Figure 3).

On the basis of the AFST results, *Δ*CPR1 had the same phenotypes as that of T-DNA mutant FOM1123 displaying low MICs to azoles (except for FLU). In contrast, the other deletion mutants (*Δ*HPG, *Δ*CPR2, *Δ*CPR3, and *Δ*CPR4) had the same phenotypes as that of the wild-type *F. oxysporum*, implying the corresponding genes were unrelated to antifungal resistance (Table 1). Accordingly, of the examined genes, only *CPR1* appears to be associated with azole resistance.

### Ergosterol Content Analysis

To clarify the regulatory effects of CPR1 on ergosterol synthesis in cell membranes, we measured the ergosterol content. Without any treatment, the ergosterol content was lower in *Δ*CPR1 than in the wild-type control. In response to the VRC treatment, the ergosterol contents of the examined strains decreased, and the ergosterol content in *Δ*CPR1 remained low (Table 4).

**Table 4:** Ergosterol content of the wild-type *F. oxysporum* and the mutant.

Strain	Ergosterol content(mg/g)	
	VRC-treated samples	Untreated samples
Wild-type	1.62 ± 0.1007^*^	3.78 ± 0.157
*Δ*CPR1	1.08 ± 0.1058^*^	2.65 ± 0.08^*^

* Data were analyzed according to ANOVA (*p*<0.005, *T* test).

### Expression Analysis of Genes Involved in Ergosterol Biosynthesis

**Figure 4:**
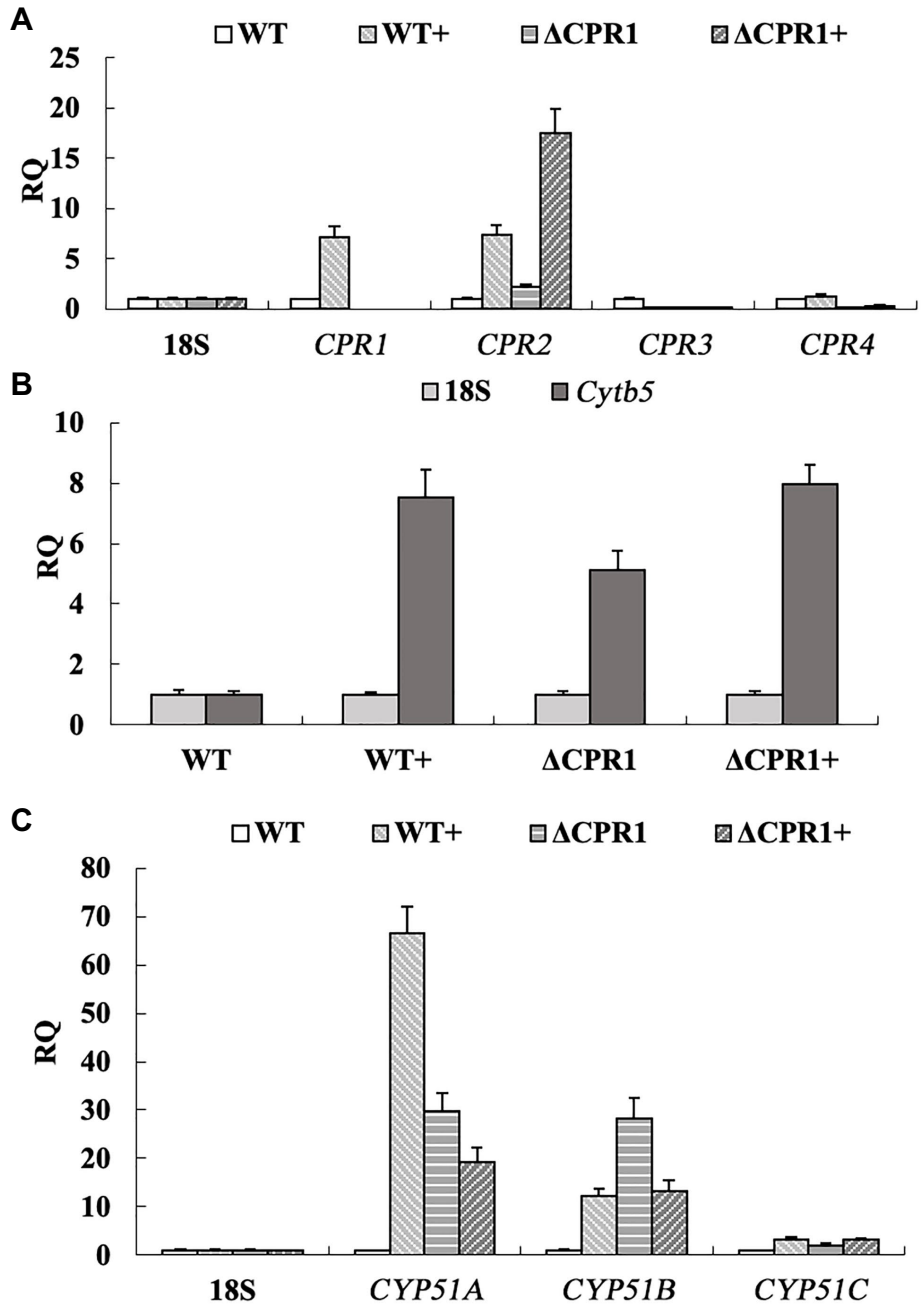
Relative expression levels of genes involved in ergosterol biosynthesis were calculated, using the 18S rRNA gene as the internal reference. **(A)** Expression of *Cpr1* and its homologs. **(B)** Expression of *Cytb5*. **(C)** Expression of *Cyp51*. “+” represents strains were treated with VRC.

To analyze the expression-level changes to the genes involved in the ergosterol biosynthesis pathway, we analyzed the relative expression of *Cpr*, *Cytb5*, and *Cyp51*. Following the VRC treatment, the *Cpr1* and *Cpr2* expression levels increased by about 7-fold, whereas *Cpr3* was almost unexpressed and *Cpr4* was unaffected in the wild-type *F. oxysporum*. In the deletion mutant *Δ*CPR1, the *Cpr2* expression level was about 2-fold higher than the corresponding level in the wild-type control, whereas *Cpr3* and *Cpr4* were almost unexpressed. When *Δ*CPR1 was treated with VRC, the expression of *Cpr2* increased by about 8.5-fold, whereas *Cpr3* and *Cpr4* expression levels were unaffected (Figure 4A).

The *Cytb5* expression level was about 5-fold higher in *Δ*CPR1 than in the wild-type *F. oxysporum*. The VRC treatment upregulated *Cytb5* expression by about 7.5-fold and 8-fold in the wild-type control and *Δ*CPR1, respectively (Figure 4B). Three homologous genes (*Cyp51A*, *Cyp51B*, and *Cyp51C*) encode proteins targeted by azole antifungal agents. These proteins may receive electrons from CPR and Cytb5. The *Cyp51A* and *Cyp51B* expression levels were about 30-fold higher in *Δ*CPR1 than in the wild-type control. The exposure to VRC increased the *Cyp51A* expression level by about 67-fold in the wild-type *F. oxysporum*. In contrast, *Cyp51A* and *Cyp51B* expression levels were downregulated by about 33 and 57%, respectively, in *Δ*CPR1. There were no significant changes to *Cyp51C* expression in any sample (Figure 4C).

## Discussion

*Fusarium* species are resistant to multiple common antifungal agents (Azor et al., 2007; Al-Hatmi et al., 2016; Tupaki-Sreepurna and Kindo, 2018; Zhao et al., 2021). Analyses of their resistance mechanisms are critical for improving clinical treatments and for preventing or mitigating antifungal resistance. Previous research confirmed *F. solani* exhibits intrinsic resistance to echinocandin, likely because of a mutation to the gene (*Fks1*) encoding the catalytic subunit of β-1,3-glucan synthase (Katiyar and Edlind, 2009; Al-Hatmi et al., 2016).

The histidine kinase III gene (*Fhk1*) in *F. oxysporum* helps regulate the Hog1-MAPK signaling pathway during stress responses. Deleting this gene leads to increased resistance to phenylpyrrole and dicarboximide fungicides (Rispail and Di Pietro, 2010). Mutations in the genes encoding β1-tubulin and β2-tubulin, which are targeted by benzimidazole fungicides, can lead to increased resistance to carbendazim in various *Fusarium* species, including *Fusarium graminearum*, *Fusarium fujikuroi*, and *Fusarium asiaticum* (Suga et al., 2011; Chen et al., 2014; Zhou et al., 2016). Hence, the antifungal resistance of *Fusarium* species is complex and regulated by multiple genes. The precise mechanisms mediating this antifungal resistance remain to be investigated.

In this study, an isolate of *F. oxysporum* with broadly resistant to commonly antifungal agents and an ATMT-based random insertional mutagenesis method was used to construct T-DNA insertion mutants. And a total of 1,450 T-DNA insertion mutants were obtained. According to the AFST results, compared with the resistant wild-type, one mutant (FOM1123) exhibited significantly increased susceptibility to azoles other than FLU (Table 1). It indicated that the gene interrupted by T-DNA insertion in this mutant might be related to the resistance of wild-type.

Bioinformatics analyses indicated that the T-DNA insertion might have affected two genes (*Hpg* and *Cpr1*; Figure 2). To confirm the involvement of these genes to the changes with azole susceptibility, the independent mutants (*Δ*HPG and *Δ*CPR1) were generated. And the AFST results revealed that only *Δ*CPR1 mutant had the same phenotypes with MICs as the T-DNA mutants (Table 1). It suggested that the *Cpr1* gene might be related to the resistance of *F. oxysporum*.

The *Cpr1* gene encodes NADPH-cytochrome P450 reductase, which is important for electron transport in various organisms. In fungi, it also participates in ergosterol biosynthesis. In an earlier study by Sutter and Loper (1989), the deletion of the CPR-encoding gene in *Saccharomyces cerevisiae* resulted in increased susceptibility to KTZ. The *F. oxysporum* genome includes four *Cpr* homologs. The independent mutants (*Δ*CPR2, *Δ*CPR3, and *Δ*CPR4) were generated in this study, and the AFST results implied these three genes do not influence antifungal resistance (Table 1). Accordingly, only *Cpr1* is associated with azole resistance in *F. oxysporum*.

Because of its function related to electron transport, CPR1 can affect the function of CYP51, which is targeted by azole antifungal agents, in the ergosterol biosynthesis pathway. Previous studies proved that deleting *CYP51A* can lead to increased susceptibility to azoles in *Magnaporthe oryzae*, *Aspergillus fumigatus*, and *F. graminearum* (Mellado et al., 2007; Yan et al., 2011). Unlike other fungi, the genomes of *Fusarium* species contain three *Cyp51* genes (*Cyp51A*, *Cyp51B*, and *Cyp51C*). Fan et al. (2013) heterologously expressed three *F. graminearum Cyp51* genes in *S. cerevisiae*. They revealed that *Cyp51A* is associated with azole susceptibility, whereas *Cyp51B* and *Cyp51C* are not. Additionally, *Cyp51A* expression is reportedly induced by ergosterol depletion. Moreover, it is responsible for the intrinsic variation in azole susceptibility. These findings imply CYP51A might be the main target regulated by CPR1.

Because both CPRs and Cytb5 can deliver electrons to CYP51s, we analyzed the expression of the corresponding genes. In this study, when the wild-type *F. oxysporum* was treated with VRC, the *Cpr1*, *Cpr2*, and *Cytb5* expression level increased (Figures 4A,B). Subsequently, the expression of *Cyp51A* and *Cyp51B* was upgraduated (Figure 4C). In response to the VRC treatment, *Cytb5* expression in *Δ*CPR1 was not significantly different from that in the wild-type control (Figure 4B), indicating *Cyp51* was unaffected by *Cytb5*. At the same time, though the expression of *Cpr2* increased (Figure 4A), the electron supply to CYP51 was insufficient owing to *Cpr1* deletion, lead to the expression of *Cyp51A* and *Cyp51B* was downgraduated than that in the wild-type (Figure 4C). Consequently, ergosterol biosynthesis was restricted, the ergosterol levels decreased significantly in *Cpr1* deletion mutant than the wild-type (Table 4), which contributed to the increase in azole susceptibility.

In conclusion, our results indicate that CPR1 plays an important role in the ergosterol biosynthesis pathway. Furthermore, it is the only NADPH-cytochrome P450 reductase related to azole resistance in *F. oxysporum*. The increased expression in the CPR1 content may ensure sufficient electrons are supplied to CYP51s for the biosynthesis of ergosterol. This may help to explain why the wild-type fungus was resistant to all tested azoles. Thus, it represents a novel therapeutic target for fungal infections.

## Data Availability Statement

The original contributions presented in the study are included in the article/supplementary material, further inquiries can be directed to the corresponding author.

## Author Contributions

DH and LW: conceptualization and design. ZF, SG, and SH: methodology and experiments. YW: data analysis. DH: original manuscript. LW: review and editing. All authors have read and approved the manuscript for publication.

## Funding

This study was supported by grants from the National Natural Science Foundation of China (81772162 and U1704283) and the Foundation of Jilin Education Committee (JJKH20211150KJ).

## Conflict of Interest

Authors SG and SH were employed by Beijing ZhongKai TianCheng Bio-technology Co. Ltd., (Beijing, China).

The remaining authors declare that the research was conducted in the absence of any commercial or financial relationships that could be construed as a potential conflict of interest.

## Publisher’s Note

All claims expressed in this article are solely those of the authors and do not necessarily represent those of their affiliated organizations, or those of the publisher, the editors and the reviewers. Any product that may be evaluated in this article, or claim that may be made by its manufacturer, is not guaranteed or endorsed by the publisher.

## Footnotes

^1^ http://blast.ncbi.nlm.nih.gov/
